# Seaweed Dietary Fiber Sodium Alginate Suppresses the Migration of Colonic Inflammatory Monocytes and Diet-Induced Metabolic Syndrome via the Gut Microbiota

**DOI:** 10.3390/nu13082812

**Published:** 2021-08-16

**Authors:** Ryuta Ejima, Masahiro Akiyama, Hiroki Sato, Sawako Tomioka, Kyosuke Yakabe, Tatsuki Kimizuka, Natsumi Seki, Yumiko Fujimura, Akiyoshi Hirayama, Shinji Fukuda, Koji Hase, Yun-Gi Kim

**Affiliations:** 1 Research Center for Drug Discovery, Faculty of Pharmacy and Graduate School of Pharmaceutical Sciences, Keio University, Tokyo 105-8512, Japan; 246911ejima@keio.jp (R.E.); akiyama.masahiro@keio.jp (M.A.); h_sato@kaigen-pharma.co.jp (H.S.); sawakotomioka@keio.jp (S.T.); kkkeeoosuyi@keio.jp (K.Y.); kimizukatatsuki@keio.jp (T.K.); natsumisoshiru826@keio.jp (N.S.); 2 Division of Biochemistry, Faculty of Pharmacy and Graduate School of Pharmaceutical Sciences, Keio University, Tokyo 105-8512, Japan; yumiko.fujimura@keio.jp (Y.F.); hase-kj@pha.keio.ac.jp (K.H.); 3 Kaigen Pharma Co., Ltd., Osaka 541-0045, Japan; 4 Institute for Advanced Biosciences, Keio University, Yamagata 997-0052, Japan; hirayama@ttck.keio.ac.jp (A.H.); sfukuda@sfc.keio.ac.jp (S.F.); 5 Transborder Medical Research Center, University of Tsukuba, Ibaraki 305-8575, Japan; 6 Intestinal Microbiota Project, Kanagawa Institute of Industrial Science and Technology, Kanagawa 210-0821, Japan

**Keywords:** gut microbiota, metabolic syndrome, inflammatory monocytes

## Abstract

Metabolic syndrome (MetS) is a multifactorial chronic metabolic disorder that affects approximately one billion people worldwide. Recent studies have evaluated whether targeting the gut microbiota can prevent MetS. This study aimed to assess the ability of dietary fiber to control MetS by modulating gut microbiota composition. Sodium alginate (SA) is a seaweed-derived dietary fiber that suppresses high-fat diet (HFD)-induced MetS via an effect on the gut microbiota. We observed that SA supplementation significantly decreased body weight gain, cholesterol levels, and fat weight, while improving glucose tolerance in HFD-fed mice. SA changed the gut microbiota composition and significantly increased the abundance of *Bacteroides*. Antibiotic treatment completely abolished the suppressive effects of SA on MetS. Mechanistically, SA decreased the number of colonic inflammatory monocytes, which promote MetS development, in a gut microbiota-dependent manner. The abundance of *Bacteroides* was negatively correlated with that of inflammatory monocytes and positively correlated with the levels of several gut metabolites. The present study revealed a novel food function of SA in preventing HFD-induced MetS through its action on gut microbiota.

## 1. Introduction

Metabolic syndrome (MetS) is a multifactorial chronic metabolic disorder characterized by obesity, insulin resistance, and hyperlipidemia, which leads to the development of type 2 diabetes. MetS affects more than one billion people worldwide and is closely associated with dietary habits, especially the consumption of high-fat foods, resulting in the accumulation of fat in various tissues due to an energy imbalance [1,2].

The gut microbiota influences various metabolic processes in the host. Many studies have reported that changes in the gut microbiota are closely linked to lipid and glucose metabolism, insulin resistance, energy metabolism, and immune function [3,4]. A high-fat diet (HFD) causes dysbiosis of the gut microbiota, in addition to its direct effects on the host metabolism [5]. This dysbiosis may promote metabolic abnormalities in the host by increasing energy harvesting, storage capacity, intestinal permeability, and inflammation [6].

Recent studies have evaluated whether targeting the gut microbiota can prevent HFD-induced MetS. For example, administration of probiotics *Lactobacillus* and *Bifidobacterium* species suppressed diet-induced obesity, which was associated with changes in gut microbiota composition and diversity [7,8,9]. In addition, supplementation of the probiotic yeast *Saccharomyces boulardii* to HFD-fed leptin-resistant obese and type 2 diabetic mice reduced body weight, fat mass, liver adiposity, and inflammatory tone, accompanied by dramatic changes in the composition of the gut microbiota [10].

Dietary fiber is a functional food that benefits the health of the host by causing specific changes in both the composition and activity of the gut microbiota [11]. Sodium alginate (SA), derived from brown algae, is a matrix polysaccharide that forms ~40% of the dry weight of the cell wall [12]. SA is used as an additive in processed foods, functioning as a food thickener, stabilizer, and gelling agent. In addition, as a dietary fiber, SA has been reported to reduce obesity and inflammation by decreasing blood glucose levels after meals, increasing fat excretion, and decreasing energy intake [13,14]. SA alters gut microbiota composition [15], which could regulate host metabolism. However, the role of SA in suppressing HFD-induced MetS through its action on the gut microbiota remains unclear. 

In this study, we demonstrate that the gut microbiota contributes to the preventive effects of SA on MetS.

## 2. Materials and Methods

### 2.1. Animals and Diets

Wild-type (WT) C57BL/6 mice were purchased from Sankyo Labo Service Corporation (Tokyo, Japan). *Ccr2*^−/−^ mice were generated by mating *Ccr2*^−/+^ mice, which were provided by Dr. Naofumi Mukaida, Kanazawa University, Ishikawa, Japan [16]. All mice were housed at the Keio University Faculty of Pharmacy, Tokyo. All experiments were approved by the ethics committee of the Keio University. Mice were fed a HFD from 9 weeks of age for 4 or 12 weeks. Compositions of the HFDs used in the study are listed in Table 1. We used two different HFDs with 60 kcal% fat and replaced cellulose with SA: a high fat purified diet with 50.0 g cellulose (D12492, HFD), or 11.3 g cellulose and 38.7 g SA (D18102802, 5% SA+HFD). The SA concentration was determined by preliminary experiments. All mice were maintained on a normal chow diet containing 22%, 23%, and 55% calories from fat, protein, and carbohydrates, respectively (CE-2, CLEA Japan, Inc., Tokyo, Japan), until the start of the experiments. At the end of the experimental period, blood was collected before euthanasia; organs such as the white adipose tissue (WAT) and liver were collected after euthanasia and weighed. The mice were fasted for approximately 12 h before euthanasia. In the experiments involving antibiotic (Abx) treatment, mice were administered drinking water containing erythromycin (200 mg/L) during HFD feeding.

### 2.2. Oral Glucose Tolerance Test (OGTT)

Mice were fasted for 6 h before the OGTT. Fasting glucose levels were measured using a OneTouch UltraVue glucometer (Johnson & Johnson, New Brunswick, NJ, USA) and LFS Quick Sensor (Johnson & Johnson). The fasted mice were orally gavaged with a 20% (*w/v*) glucose solution (2 g/kg body weight (BW)). Thereafter, blood glucose levels were measured at 15, 30, 60, and 120 min.

### 2.3. Total Blood Cholesterol

Blood was collected from the mice and centrifuged at 2000× *g* at 4 °C for 15 min with 1 µL of heparin sodium. The plasma supernatant was stored at −80 °C. Plasma cholesterol levels were measured using the FUJI DRI-CHEM SLIDE TCHO-PIII, FUJI DRI-CHEM SLIDE TG-PIII, and FUJI DRI-CHEM SYSTEM 7000i (Fujifilm, Tokyo, Japan).

### 2.4. Histological Analysis

Liver tissue samples were fixed in a 10% formalin neutral buffer solution (Mildform 10N, Wako Pure Chemical Industries, Ltd., Osaka, Japan). The fixed samples were embedded in paraffin, and the sections cut into 3 μm were deparaffinized, rehydrated, stained with hematoxylin (Agilent Technologies, Inc., Miami, FL, USA) and eosin (Wako, Osaka, Japan), and mounted using Mount-Quick (Daido Sangyo Co., Ltd., Tokyo, Japan).

### 2.5. Quantification of Hepatic Lipid Droplets in Hematoxylin–Eosin (H&E)-Stained Sections

To measure the hepatic lipid droplets, images of the sections from each sample were binarized using the imager R package (version 0.42.10, https://cran.r-project.org/), and the percentage of white area was calculated.

### 2.6. Sequencing and Processing of Bacterial 16S rRNA Genes in Fecal DNA

Bacterial DNA was extracted from the feces as described previously [17]. The V3–V4 region of the 16S gene was amplified using the KAPA HiFi HotStart ReadyMix (Nippon Genetics Co., Ltd., Tokyo, Japan) with the primer set (forward: 5ʹ-TCGTCGGCAGCGTCAGATGTGTATAAGAGACAGCCTACGGGNGGCWGCAG-3ʹ; reverse, 5ʹ-GTCTCGTGGGCTCGGAGATGTGTATAAGAGACAGGACTACHVGGGTATCT AATCC-3ʹ) and purified using AMPure XP (Beckman Coulter, Inc., Brea, CA, USA). Different index sequences were added to each DNA sample using the Nextera XT index kit (Illumina, Inc., San Diego, CA, USA). Then equal amounts of each amplified DNA sample were mixed and sequenced using a MiSeq System (Illumina, San Diego, CA, USA) with a 2 x 300-base-pair protocol (Miseq Reagent Kit V3, 600 cycles, Illumina). Sequencing data were analyzed using Qiime2 (version 2021.4) [18]. The primer region was trimmed from the raw sequences by employing Cutadapt in the Qiime2 plugin (https://doi.org/10.14806/ej.17.1.200, accessed on 27 May 2021). The primer-free sequences were processed with DADA2 [19] to remove PhiX contamination and PCR chimeras, trim reads, correct errors, and merge read pairs, and to obtain representative amplicon sequence variant (ASV) sequences. For each ASV-representative sequence, BLAST [20] was used to assign taxonomy based on the SILVA database (version 138) [21]. The representative ASV sequences and their abundances were extracted by feature table [22]; then, the compositional data were converted, and principal coordinate analysis (PCoA) was performed to analyze beta diversity using an unweighted UniFrac metric calculated by QIIME2.

### 2.7. Isolation of Colonic Lamina Propria Cells

Colonic lamina propria cells were isolated as previously described [23] with some modifications. The colons were opened longitudinally and washed vigorously with HBSS (Nacalai Tesque, Inc., Kyoto, Japan). Each colonic tissue was cut into four segments and stirred in HBSS containing 1 mM dithiothreitol, 20 mM EDTA, 10 µM DTT, and 12.5 mM HEPES (Nacalai Tesque) at 37 °C for 20 min two times. The tissues were then minced and dissociated with RPMI1640 (Nacalai Tesque) containing 0.125 mg/mL DNase I (Merck, Darmstadt, Germany), 0.2 U/mL Liberase TM (Roche Diagnostics, Mannheim, Germany), 2% NBCS (Thermo Fisher Scientific, Waltham, MA, USA), 100 U/mL penicillin, 100 µg/mL streptomycin (Nacalai Tesque), and 20 mM HEPES at 37 °C for 40 min. After filtering, the cell suspensions were washed with HBSS and subjected to flow cytometry analysis.

### 2.8. Flow Cytometry

Colonic lamina propria cells were incubated with the anti-mouse CD16/CD32 antibody (93; BioLegend, Inc., San Diego, CA, USA) to block Fc receptors and then stained using antibodies conjugated with eF450, Brilliant Violet 421 (BV), BV 510, FITC, Alexa Fluor 488, phycoerythrin (PE), PerCP-Cyanine5.5, PE-CF594, PE-Cy7, allophycocyanin (APC), or APC-Cy7.GATA3 (L50-823). The Ly6G (1A8), RORγt (Q31-378), Siglec-F (E50-2440), CD11c (HL3), and Ly6C (AL-21) antibodies were obtained from BD Biosciences (San Jose, CA, USA) CD45 (30-F11), whereas the CD3ε (145-2C11), F4/80 (BM1), CX_3_CR1 (SA011F11), T-bet (4B10), and F4/80 (BM8) antibodies were purchased from BioLegend. The MHC class II (M5/114.15.2), CD11b (M1/70), Foxp3 (FJK-16s), and CD4 (GK1.5) antibodies were acquired from eBioscience (San Diego, CA, USA). 7-AAD (BioLegend), or the Fixable Viability Stain 780 (BD Biosciences) was added to the cell suspension to label dead cells. Stained cells were analyzed using MACSQuant (Miltenyi Biotec, Bergisch Gladbach, Germany).

### 2.9. CE-TOFMS Metabolome Analysis

Fecal samples were freeze-dried and disrupted by vigorous shaking at 1500 rpm for 10 min with four 3 mm zirconia beads using a Shake Master NEO (Bio Medical Science, Tokyo, Japan). Fecal samples (10 ± 0.5 mg) were homogenized with 500 μL MeOH containing internal standards (20 μM each of methionine sulfone and D-camphor-10-sulfonic acid) and 100 mg of 0.1 mm and four 3 mm zirconia/silica beads (Biospec Products, Bartlesville, OK, USA). After vigorous shaking (1500 rpm for 5 min) using a Shake Master NEO (Bio Medical Science), 200 μL of Milli-Q water and 500 μL of chloroform were added, and the mixture was stirred again. After centrifugation at 4600× *g* and 4 °C for 15 min, the supernatant was transferred to a centrifugal filter tube with a 5 kDa limit. The filtrate was concentrated via centrifugation at 40 °C and reconstituted with 40 μL of Milli-Q water. Ionic metabolites were analyzed using CE-TOFMS in both the positive and negative modes [24]. All CE-TOFMS experiments were performed using an Agilent CE capillary electrophoresis system (Agilent Technologies). To identify peak annotation and quantification, the obtained data were processed using an in-house software (MasterHands) [25]. Principal component analysis (PCA) was based on Euclidean distances.

### 2.10. Statistical Analyses

Statistical analyses were performed using the GraphPad Prism software (version 9.1.2; GraphPad Software Inc.). Differences between two groups were evaluated using the D’Agostino and Pearson test and F-test, followed by the Student’s *t*-test, Welch’s *t*-test, or the Mann–Whitney U test. Comparisons of more than two groups were performed using two-way repeated measures ANOVA, followed by Šidák corrections for multiple comparisons. Spearman’s correlation analysis was performed on significantly changed gut microbiota and inflammatory monocytes (IMϕ or anti-inflammatory macrophages (AMϕ) using R. Differences were considered statistically significant at *p* < 0.05.

## 3. Results

### 3.1. Dietary Supplementation with SA Suppresses HFD-Induced MetS

**Figure 1:**
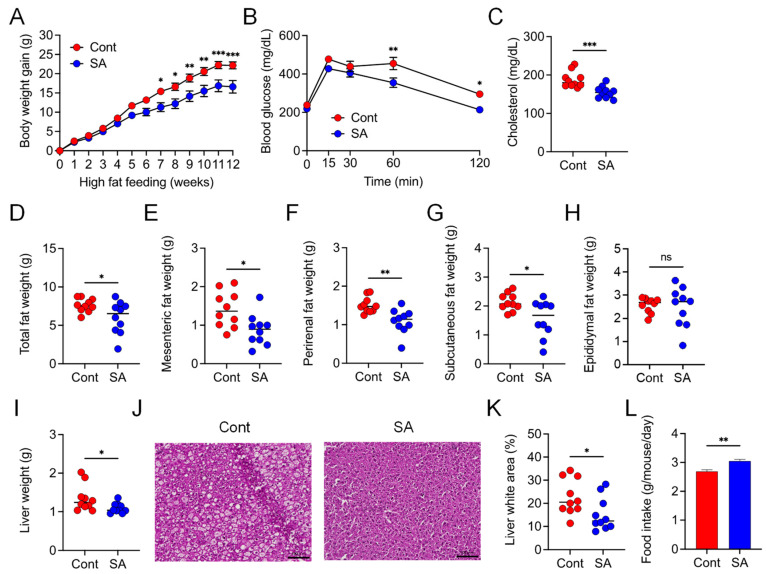
Dietary treatment with sodium alginate (SA) suppressed high fat diet (HFD)-induced metabolic syndrome (MetS). Mice were fed a HFD supplemented with or without 5% SA for 12 weeks (*n* = 10 mice per group). (**A**) Body weight gain. (**B**) Blood glucose levels, as measured via an oral glucose tolerance test (OGTT). (**C**) Cholesterol levels in plasma. (**D**) Total white adipose tissue (WAT) weight. (**E**) Mesenteric WAT weight. (**F**) Perirenal WAT weight. (**G**) Subcutaneous WAT weight. (**H**) Epididymal WAT weight. (**I**) Liver weight. (**J**) Representative images of lipid droplet-containing areas (white areas) and (**K**) percentage of lipid droplet-containing areas in hematoxylin and eosin (H&E)-stained liver sections. Scale bar, 100 μm. (**L**) Daily food intake of the mice. Each dot represents an individual mouse, and the horizontal bars indicate mean values. Statistical significance was assessed by unpaired Student’s *t*-test in (**C,E,F,K,L**); Welch’s *t*-test in (**D,G,H,I**); and two-way analysis of variance (ANOVA) with Sidak’s multiple comparisons test in (**A,B**). * *p* < 0.05; ** *p* < 0.01; *** *p* < 0.001; n.s., not significant; Cont: HFD diet; SA: HFD diet with 5% sodium alginate.

We first verified the inhibitory effect of SA on diet-induced MetS. Compared with controls, mice that were fed a HFD and treated with SA for 12 weeks exhibited reduced body weight gain and plasma cholesterol levels and improved glucose tolerance, as assessed by the OGTT (Figure 1A–C). In addition, the mesenteric, perirenal, subcutaneous, and total fat weights were significantly lower in the SA-treated group than in the controls (Figure 1D–G); however, there was no significant difference in the epididymal fat weight (Figure 1H). Liver weights were also lower in the SA-treated group than in the control group (Figure 1I). Furthermore, histological analysis revealed that the lipid droplet area in the liver was reduced after SA treatment (Figure 1J,K). Meanwhile, food intake was slightly increased in the SA-treated group than in controls (Figure 1L). Collectively, these results indicate that supplementing the diet with SA ameliorates HFD-induced MetS.

### 3.2. Suppression of Diet-Induced Obesity by SA Depends on the Gut Microbiota

**Figure 2:**
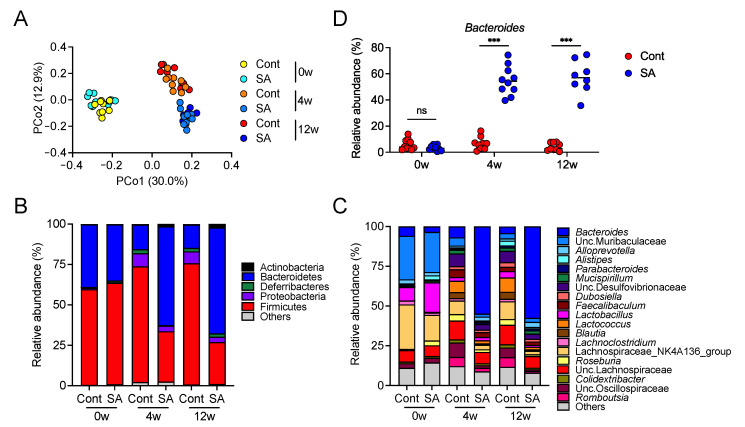
Dietary treatment with sodium alginate (SA) altered the gut microbiota composition of high fat diet (HFD)-fed mice. Mice were fed a HFD supplemented with or without 5% SA for 0, 4, or 12 weeks (*n* = 8–10 mice per group). (**A**) Principal component analysis (PCoA) of the microbiome in fecal samples was performed using unweighted UniFrac. (**B**,**C**) Relative abundance of amplicon sequence variants (ASVs). The different colors correspond to different bacterial phyla (**B**) and genera (**C**), as indicated. (**D**) Relative abundance of *Bacteroides* in the fecal samples. Each dot represents an individual mouse, and the horizontal bars indicate mean values. Statistical significance was assessed by Welch’s *t*-test (**D**). *** *p* < 0.001; n.s., not significant. Cont: HFD diet; SA: HFD diet with 5% sodium alginate.

**Figure 3:**
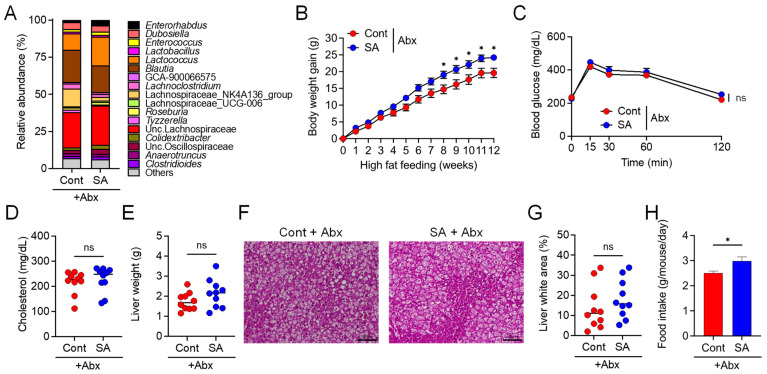
Gut microbiota was involved in the sodium alginate (SA)-mediated suppression of high fat diet (HFD)-induced MetS. Mice were fed a HFD supplemented with or without 5% SA for 12 weeks and subjected to antibiotic (Abx) treatment (*n* = 10 mice per group). (**A**) Relative abundance of ASVs in feces of mice maintained on a HFD supplemented with or without 5% SA for 4 weeks. The different colors correspond to different bacterial genera, as indicated. (**B**) Body weight gain. (**C**) Blood glucose levels, as measured via an OGTT. (**D**) Plasma cholesterol levels. (**E**) Liver weight. (**F**) Representative images of lipid droplet-containing areas (white areas) and (**G**) percentage of lipid droplet-containing areas in H&E stained liver sections. Scale bar, 100 μm. (**H**) Daily food intake of the mice. Each dot represents an individual mouse, and the horizontal bars indicate mean values. Statistical significance was assessed by an unpaired Student’s *t*-test in (**E**,**G**), Mann–Whitney U-test in (**D**,**H**), and two-way analysis of variance (ANOVA) with Sidak’s multiple comparisons test in (**B**,**C**). * *p* < 0.05; n.s., not significant. Cont: HFD diet; SA: HFD diet with 5% sodium alginate; Abx: erythromycin treatment.

Subsequently, we examined whether the inhibitory effects of SA on MetS depended on the gut microbiota. Analysis of the 16S ribosomal RNA (rRNA) genes of the mouse fecal microbiota revealed that HFD intake altered the gut microbiota composition. Moreover, these HFD-dependent changes in the gut microbiota composition differed between the control and SA-treated groups 4 weeks as well as 12 weeks after HFD intake (Figure 2A–C). HFD intake increased the abundance of Firmicutes and Proteobacteria and decreased the abundance of Bacteroidetes. Supplementation of HFD-fed mice with SA dramatically increased the abundance of Bacteroidetes and prevented the increase in Firmicutes and Proteobacteria (Figure 2B). Furthermore, supplementation with SA increased the abundance of the *Bacteroides* genus (Figure 2C). These results suggest that SA alters the gut microbiota composition and may ameliorate the effect of SA on HFD-induced MetS. To investigate whether the gut microbiota is required for the suppressive effects of SA, mice were maintained on a HFD, supplemented with or without SA and treated with Abx to deplete the microbiota. Gut microbiota composition was comparable between the control and SA-treated groups, and *Bacteroides* were undetectable following treatment with erythromycin (Figure 3A). SA treatment did not suppress but instead promoted body weight gain in response to HFD (Figure 3B). In addition, SA did not improve glucose tolerance (Figure 3C) or reduce plasma cholesterol levels (Figure 3D), liver weight (Figure 3E), or the lipid droplet area in the liver (Figure 3F,G). Food intake was slightly increased in the SA-treated group than in controls (Figure 3H). These results demonstrate that the preventive effect of SA on HFD-induced MetS depends on the gut microbiota.

### 3.3. SA Reduces Colonic Inflammatory Monocytes in a Gut Microbiota-Dependent Manner

**Figure 4:**
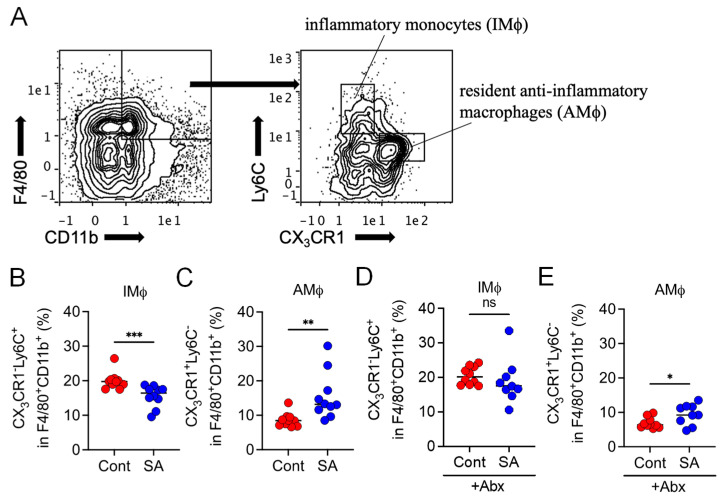
Dietary treatment with sodium alginate (SA) reduced the abundance of inflammatory monocytes in the colon by altering the gut microbiota composition. Mice were fed a HFD supplemented with or without 5% SA for 4 weeks in the absence (**B**,**C**) or presence (**D**,**E**) of antibiotic (Abx) treatment (*n* = 9 or 10 mice per group). The expression levels of CX_3_CR1 and Ly6C in CD11b^+^F4/80^+^ cells in the colon were analyzed via flow cytometry. (**A**) Representative flow cytometry images and frequencies of (**B**,**D**) CD11b^+^F4/80^+^CX_3_CR1^low^Ly6C^+^ (inflammatory monocytes, IMϕ) and (**C**,**E**) CD11b^+^F4/80^+^CX_3_CR1^high^Ly6C^−^ cells (resident anti-inflammatory macrophages, AMϕ). Each dot represents an individual mouse, and the horizontal bars indicate mean values. Statistical significance was assessed by Mann–Whitney U-test in (**B**–**D**), and unpaired Student’s *t*-test in (**E**). * *p* < 0.05; ** *p* < 0.01; *** *p* < 0.001; n.s., not significant. Cont: HFD diet; SA: HFD diet with 5% sodium alginate; Abx: erythromycin treatment.

Next, we elucidated the mechanism by which SA suppresses MetS. Since the influx of inflammatory monocytes into the intestines of HFD-fed mice has been associated with metabolic dysfunction [26], we hypothesized that SA supplementation influences the macrophage population. To test this hypothesis, cells were isolated from colonic tissues, and the macrophage population was evaluated. Because SA had already changed the gut microbiota composition in 4 weeks when the difference in the weight gain was not observed after HFD intake, we assessed the cell population at this time point. Compared to the control mice, SA-treated mice displayed a decreased abundance of CD11b^+^F4/80^+^CX_3_CR1^low^Ly6C^+^ cells (IMϕ) and increased abundance of CD11b^+^F4/80^+^CX_3_CR1^high^Ly6C^−^ cells (AMϕ) in the colon (Figure 4A,B). We next assessed whether alterations in the macrophage population due to SA were dependent on the gut microbiota. Abx treatment abolished the decrease in abundance of colonic IMϕ while still increasing the abundance of colonic AMϕ after SA supplementation (Figure 4C). The chemokine ligand 2 (CCL2)–CC chemokine receptor type 2 (CCR2) axis plays a critical role in the egress of Ly6C^+^ inflammatory monocytes from the bone marrow (BM) to the gut mucosa and is involved in insulin resistance [26]. Therefore, we assessed whether SA controls MetS via the CCL2–CCR2 axis. Although treatment with SA significantly increased the relative abundance of *Bacteroides* in the intestine of *Ccr2*^−/−^ mice (Figure S1A), no significant differences were observed in body weight gain (Figure S1B), glucose tolerance (Figure S1C), blood cholesterol levels (Figure S1D), or fat weight (Figure S1E–J) in SA-treated mice compared to the control group 12 weeks after HFD intake. Food intake levels were comparable between the SA-treated and non-treated groups (Figure S1K). These results indicate that SA confers protective effects against MetS via the CCL2–CCR2 axis.

In addition to macrophages, we also monitored another population of immune cells in the intestine. The abundance of neutrophils (CD11b^+^Ly6G^+^Ly6C^+^, Figure S2A) and monocyte-derived dendritic cells (CD11c^+^CD11b^+^MHCII^+^Ly6C^+^, Figure S2B), which differentiate from Ly6C^+^ IMϕ [27], was significantly lower, while that of conventional dendritic cells (CD11c^+^CD11b^+^MHCII^+^Ly6C^−^, Figure S2C) [28] was higher in the colonic tissues from the SA-treated mice than those from the controls. The abundance of eosinophils (CD11b^+^F4/80^+^SiglecF^+^) was comparable between the colonic tissues from SA-treated mice and those from the controls (Figure S2D). These differences were not observed after Abx treatment (Figure S2E–G). No difference was observed in the population of CD4^+^ T cell subsets, namely, T helper type-1, -2, and -17 cells, and T-regulatory cells, between the control and SA-treated groups (Figure S3).

Collectively, we concluded that SA intake regulates the accumulation of IMϕ in the intestine, which is a key factor that prevents MetS by changing the gut microbiota composition.

### 3.4. Increase in Bacteroides Is Negatively Correlated with the Abundance of Colonic Inflammatory Monocytes

**Figure 5:**
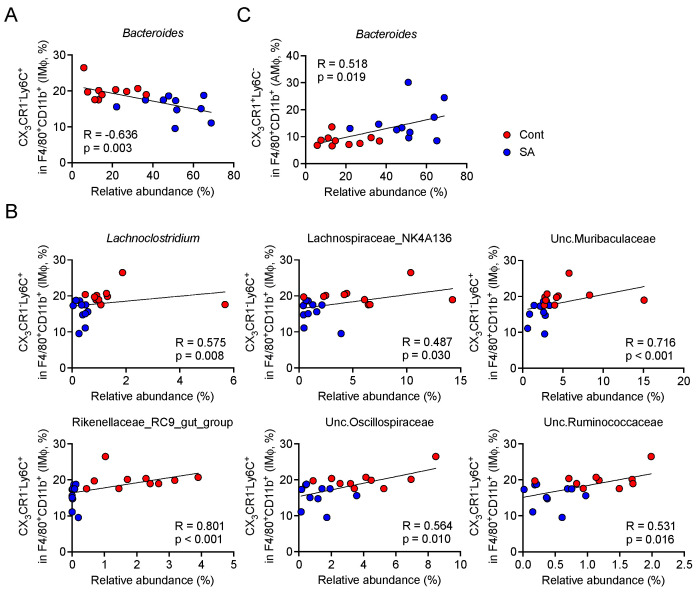
*Bacteroides* were negatively correlated with the abundance of inflammatory monocytes in the colon. Mice were fed a HFD supplemented with or without 5% sodium alginate (SA) for 4 weeks (*n* = 10 mice per group). Spearman’s rank correlation coefficient was used to analyze the correlation between the relative abundance of gut microbiota and the percentage of colonic macrophages. (**A**,**B**) Scatter plots showing the (**A**) negative or (**B**) positive correlation between the gut microbiota and CD11b^+^F4/80^+^CX_3_CR1^low^Ly6C^+^ cells in the colon. (**C**) Scatter plots showing the positive correlation between gut microbiota and CD11b^+^F4/80^+^CX_3_CR1^high^Ly6C^−^ cells in the colon. Cont: HFD diet; SA: HFD diet with 5% sodium alginate; R: spearman correlation coefficient; *p*: *p*-value.

We next explored which gut bacteria correlated with the abundance of colonic IMϕ. We observed that the SA-intake-dependent increase in *Bacteroides* was negatively correlated with IMϕ (Figure 5A), whereas that in the members of the Lachnospiraceae, Rikenellaceae, Ruminococcaceae, Muribaculaceae, and Oscillospiraceae families and the *Lachnoclostridium* genus was positively correlated with IMϕ (Figure 5B). In contrast, only *Bacteroides* showed a weak and positive correlation with the abundance of colonic AMϕ (Figure 5C).

### 3.5. SA Increases Metabolites That Are Positively Correlated with the Abundance of Bacteroides

**Figure 6:**
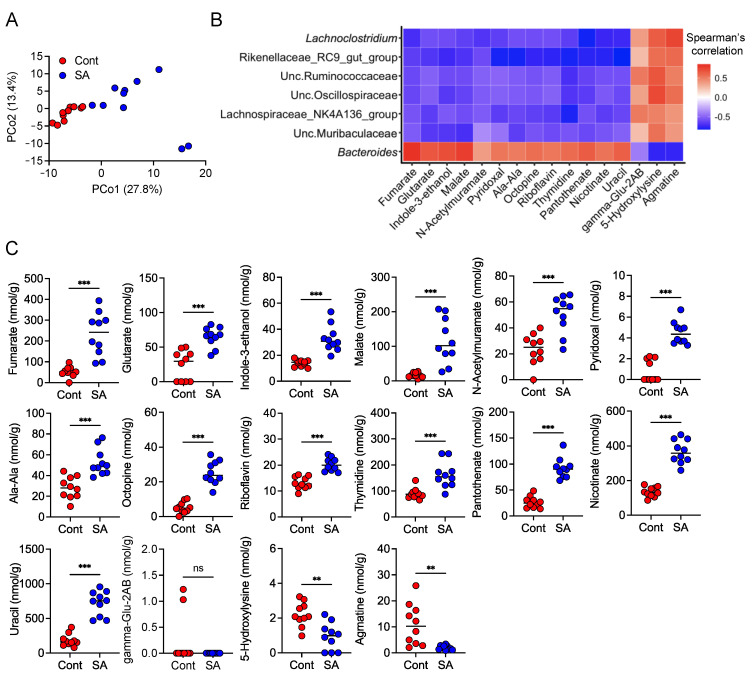
Dietary treatment with sodium alginate (SA) increased the levels of metabolites that correlated positively with the abundance of *Bacteroides*. Metabolites in mouse feces were analyzed by CE-TOFMS. Metabolome analyses were perFigure 4. weeks after feeding the mice HFD supplemented with or without 5% SA (*n* = 10 mice per group). (**A**) PCoA for the fecal samples was performed using unweighted UniFrac. (**B**) Heat map shows correlations between gut bacteria abundance and levels of fecal metabolites by Spearman’s correlation analysis. Red and blue colors denote the positive and negative correlation values, respectively. (**C**) Measurement of the levels of metabolites in the feces of mice maintained on a HFD supplemented with or without 5% SA. Each dot represents an individual mouse, and the horizontal bars indicate mean values. Statistical significance in (**C**) was assessed by unpaired Student’s *t*-test for glutarate, N-acetylmurate, pyridoxal, Ala-Ala, riboflavin, and 5-hydroxylysine; Welch’s *t*-test for fumarate, indole-3-ethanol, malate, octopine, uracil, and agmatine; and Mann–Whitney U-test for thymidine, pantothenate, nicotinate, and gamma-Glu-2AB. ** *p* < 0.01; *** *p* < 0.001; n.s., not significant. Cont: HFD diet, SA: HFD diet with 5% sodium alginate.

Finally, we performed fecal metabolomics to identify metabolites that were positively correlated with the abundance of *Bacteroides*. PCoA revealed that SA treatment significantly altered the composition of the fecal metabolites 4 weeks after HFD intake (Figure 6A). The abundance of *Bacteroides*, which was negatively correlated with the abundance of colonic IMϕ, showed a positive correlation with the fecal concentrations of fumarate, glutarate, indole-3-ethanol, malate, N-acetylmurate, pyridoxal, L-alanyl-L-alanine, octopine, riboflavin, thymidine, pantothenate, nicotinate, and uracil (Figure 6B). In contrast, the abundance of Lachnospiraceae, Rikenellaceae, Ruminococcaceae, Muribaculaceae, Oscillospiraceae, and *Lachnoclostridium* spp., which were positively correlated with the abundance of colonic IMϕ, showed a negative correlation with these metabolites and a positive correlation with gamma-Glu-2AB, 5-hydroxylysine, and agmatine (Figure 6B). Interestingly, the abundance of *Bacteroides* was negatively correlated with gamma-Glu-2AB, 5-hydroxylysine, and agmatine (Figure 6B). Consistently, SA intake increased the levels of metabolites that were positively correlated with the abundance of *Bacteroides* (Figure 6C). SA supplementation decreased the levels of metabolites that were negatively correlated with the abundance of *Bacteroides*, except gamma-Glu-2AB (Figure 6C).

## 4. Discussion

The present study demonstrated that the preventive effect of the seaweed dietary fiber SA on HFD-induced MetS was attributed to the gut microbiota. In addition, we found that SA supplementation reduced the abundance of colonic Ly6C^+^ inflammatory monocytes by changing the gut microbiota composition. The egress of Ly6C^hi^ monocytes from the BM to the intestine is dependent on the chemokine receptor CCR2 [27]. In the steady state, intestinal Ly6C^hi^ monocytes recruited from the BM give rise to CX_3_CR1^hi^ resident macrophages, which retain their anti-inflammatory signature [27]. In contrast, during inflammation, the newly recruited Ly6C^hi^ monocytes do not mature completely into anti-inflammatory CX_3_CR1^hi^ macrophages and retain their ability to produce high levels of proinflammatory cytokines [27,29]. Abx treatment prevented the decrease in the abundance of Ly6C^hi^ IMϕ caused by SA supplementation while still increasing the abundance of CX_3_CR1^hi^ AMϕ. These results suggest that SA inhibits the migration of IMϕ into the intestine via the gut microbiota but promotes the shift from IMϕ to AMϕ in a partially dependent or independent manner. Consistent with this notion, we identified several gut bacteria that were positively or negatively correlated with the abundance of IMϕ, while *Bacteroides* were the only bacteria that correlated with the abundance of AMϕ.

HFD-induced MetS was suppressed after treatment with SA in WT mice, while the effect of SA was abolished in *Ccr2*^–/–^ mice despite the significant increase in the abundance of *Bacteroides*. These results indicate that the CCL2–CCR2 axis is a critical factor regulating the preventive effects of SA against MetS. However, *Ccr2*^–/–^ mice that were fed a HFD had significantly less severe MetS than the WT mice. *Ccr2* deficiency has been reported to affect the nervous system and reduce food intake in mice [30]. Therefore, it is possible that the protective effects of SA on MetS in *Ccr2*^–/–^ mice were abrogated by the insufficient intake of calories. Thus, macrophage-specific Ccr2 knockout mice will reveal more clearly whether preventive effects of SA on diet-induced MetS are dependent on IMϕ.

The abundance of *Bacteroides* correlated negatively with the abundance of IMϕ, suggesting that *Bacteroides* could contribute to the control of IMϕ accumulation in the colon and suppress HFD-induced MetS. Indeed, the relative abundance of *Bacteroides* increased with SA intake. In addition, treatment with erythromycin eliminated *Bacteroides* and abolished the preventive effect of SA on HFD-induced MetS. Furthermore, it decreased the abundance of IMϕ. Conversely, Lachnospiraceae, Rikenellaceae, Ruminococcaceae, Muribaculaceae, and Oscillospiraceae, which were positively correlated with the abundance of IMϕ, could promote HFD-induced gut inflammation and MetS.

The gut metabolites fumarate, glutarate, indole-3-ethanol, malate, N-acetylmurate, pyridoxal, L-alanyl-L-alanine, octopine, riboflavin, thymidine, pantothenate, nicotinate, and uracil, which were positively correlated with *Bacteroides*, may regulate colonic IMϕ accumulation. In contrast, gamma-Glu-2AB, 5-hydroxylysine, and agmatine, which were negatively correlated with *Bacteroides,* might have an opposite effect. However, we performed only correlation analysis among IMϕ, *Bacteroides*, and gut metabolites. Several reports have shown that *Bacteroides* species, including *B. acidifaciens* [31], *B. uniformis* [32,33], and *B. xylanisolvens* [34], ameliorate host metabolic dysfunction in mice fed a HFD. Hence, future studies are required to confirm the effects of *Bacteroides* and their correlated metabolites on colonic IMϕ accumulation and HFD-induced MetS.

Taken together, the present study revealed that SA suppressed colonic infiltration of IMϕ and diet-induced MetS in gut microbiota-dependent manner. These findings indicate that SA changes the gut environment, including gut microbiota composition, metabolites, and gut mucosal immunity, as a consequence, which modulates host metabolism. Thus, we propose a new concept that the use of specific dietary fiber can suppress gut inflammation to prevent diet-induced MetS.

## Figures and Tables

**Table 1:** Composition of the diet used for the study.

**Product #**	**D12492**		**D18102802**	
	**g%**	**kcal%**	**g%**	**kcal%**
Protein	26	20	26	20
Carbohydrate	26	20	26	20
Fat	35	60	35	60
Total		100		100
kcal/g	5.2		5.2	
**Ingredient**	**g**	**kcal**	**g**	**kcal**
Casein	200	800	200	800
L-cystine	3	12	3	12
Maltodextrin 10	125	500	125	500
Sucrose	68.8	275	68.8	275
Cellulose	50	0	11.3	0
Soybean oil	25	225	25	225
Lard	245	2205	245	2205
Mineral mix, S10026	10	0	10	0
Dicalcium phosphate	13	0	13	0
Calcium carbonate	5.5	0	5.5	0
Potassium citrate, 1 H_2_O	16.5	0	16.5	0
Vitamin mix, V10001	10	40	10	40
Choline bitartrate	2	0	2	0
Sodium alginate	0	0	38.7	0
FD&C Yellow Dye #5	0	0	0.025	0
FD&C Red Dye #40	0	0	0	0
FD&C Blue Dye #1	0.05	0	0.025	0
Total	773.85	4057	773.85	4057

## Data Availability

The data presented in this study will be made available by the corresponding author upon request.

## Supplementary Materials

The following are available online at https://www.mdpi.com/article/10.3390/nu13082812/s1, Figure S1: Dietary treatment with sodium alginate (SA) did not suppress high fat diet (HFD)-induced MetS in Ccr2^–/–^ mice, Figure S2: Dietary treatment with sodium alginate (SA) influenced the population of innate immune cells in the gut of high fat diet (HFD)-fed mice, Figure S3: Dietary treatment with sodium alginate (SA) did not alter the population of CD4^+^ T cells in the colon of high fat diet (HFD)-fed mice.
