# Supplementary material for: Seaweed Dietary Fiber Sodium Alginate Suppresses the Migration of Colonic Inflammatory Monocytes and Diet-Induced Metabolic Syndrome via the Gut Microbiota
Source: Nutrients. 2021 Aug 16;13(8):2812. doi: 10.3390/nu13082812 (PMC8401899; doi:10.3390/nu13082812)
Supplement: Supplementary file 1 [file nutrients-13-02812-s001.zip › nutrients-1343719-supplementary.pdf]

Supplemental Figures

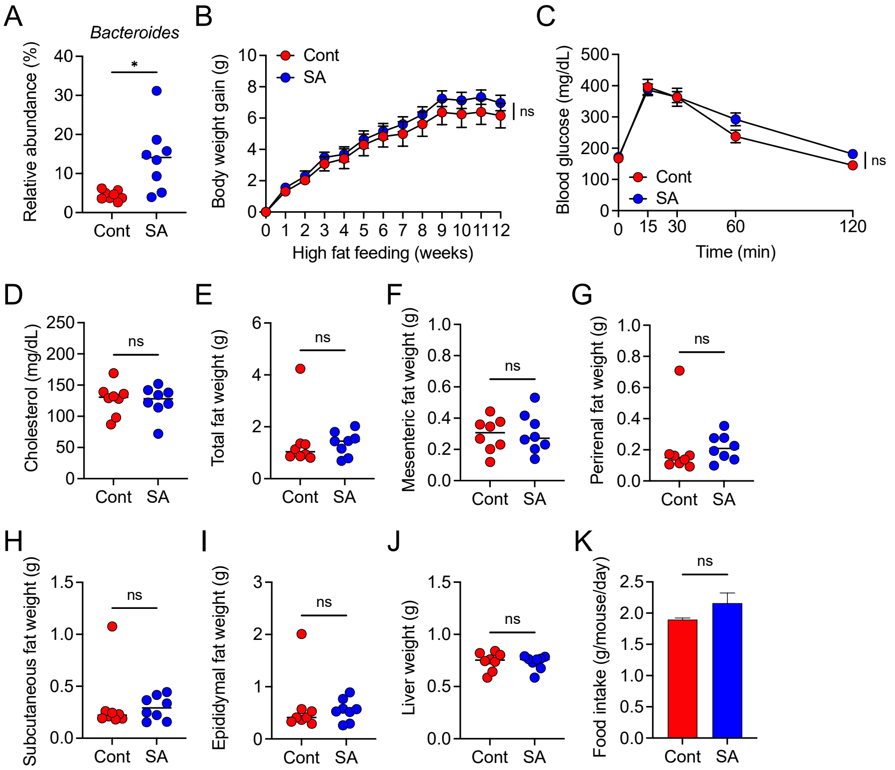

Figure S1. Dietary treatment with sodium alginate (SA) did not suppress high fat diet (HFD)-induced MetS in *Ccr2*$^{-/-}$ mice. *Ccr2*$^{-/-}$ mice were fed a HFD supplemented with or without 5% SA for 12 weeks (n = 8 mice per group). (**A**) Relative abundance of *Bacteroides* in the fecal samples of *Ccr2*$^{-/-}$ mice, maintained on a HFD supplemented with or without 5% SA for 4 weeks. (**B**) Body weight gain. (**C**) Blood glucose levels as measured via an OGTT. (**D**) Plasma cholesterol levels. (**E**) Total WAT weight. (**F**) Mesenteric WAT weight. (**G**) Perirenal WAT weight. (**H**) Subcutaneous WAT weight. (**I**) Epididymal WAT weight. (**J**) Liver weight. (**K**) Daily food intake of the mice. Each dot represents an individual mouse, and the horizontal bars indicate mean values. Statistical significance was assessed by Welch's *t*-test in (**A**,**K**); Mann–Whitney U-test in (**D**,**E**–**J**); unpaired Student's *t*-test in (**F**); and two-way ANOVA with Sidak's multiple comparisons test in (**B**,**C**). * $p < 0.05$; n.s., not significant. Cont: HFD diet, SA: HFD diet with 5% sodium alginate.

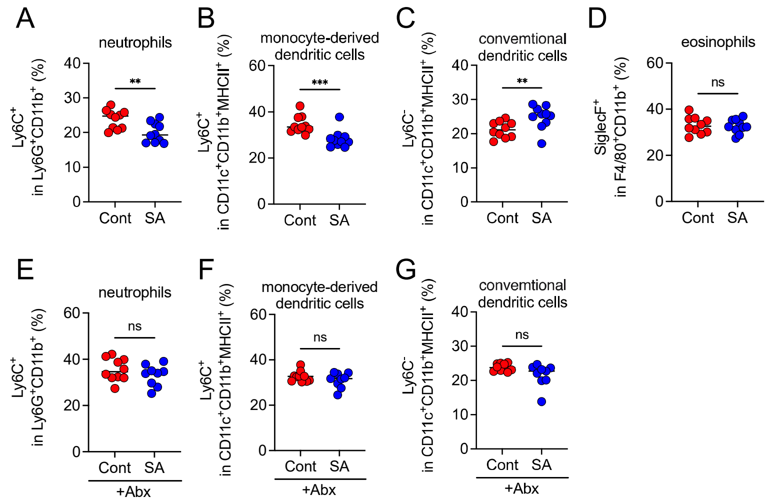

Figure S2. Dietary treatment with sodium alginate (SA) influenced the population of innate immune cells in the gut of high fat diet (HFD)-fed mice. Frequency of (**A**) neutrophils (CD11b$^+$Ly6G$^+$Ly6C$^+$), (**B**) monocyte-derived dendritic cells (CD11c$^+$CD11b$^+$MHCII$^+$Ly6C$^+$), (**C**) conventional dendritic cells (CD11c$^+$CD11b$^+$MHCII$^+$Ly6C$^-$), and (**D**) eosinophils (CD11b$^+$F4/80$^+$SiglecF$^+$) in the colon of mice maintained on a HFD supplemented with or without 5% SA for 4 weeks. Frequency of (**E**) neutrophils, (**F**) monocyte-derived dendritic cells, and (**G**) conventional dendritic cells in the colons of mice maintained on a HFD supplemented with or without 5% SA for 4 weeks and subjected to Abx treatment (n = 10 mice per group). Each dot represents an individual mouse, and the horizontal bars indicate mean values. Statistical significance was assessed by unpaired Student's *t*-test in (**A**,**D**–**F**); and Mann–Whitney U-test in (**B**,**C**,**G**). * $p < 0.05$; ** $p < 0.01$; *** $p < 0.001$; n.s., not significant. Cont: HFD diet, SA: HFD diet with 5% sodium alginate. Abx: erythromycin treatment.

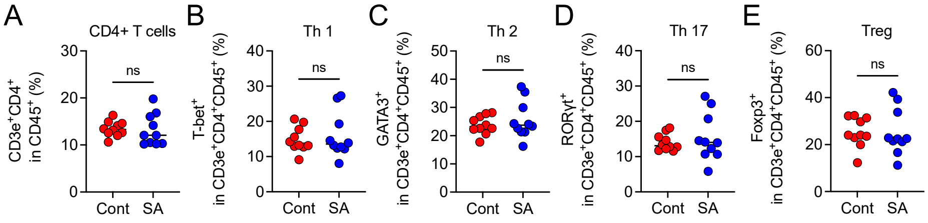

Figure S3. Dietary treatment with sodium alginate (SA) did not alter the population of CD4$^+$ T cells in the colon of high fat diet (HFD)-fed mice. Frequency of (A) CD4$^+$ T helper (Th) cells (CD45$^+$CD3e$^+$CD4$^+$), (B) Th1 cells (CD45$^+$CD3e$^+$CD4$^+$T-bet$^+$), (C) Th2 cells (CD45$^+$CD3e$^+$CD4$^+$GATA3$^+$), (D) Th17 cells (CD45$^+$CD3e$^+$CD4$^+$RORγt$^+$), and (E) Treg (CD45$^+$CD3e$^+$CD4$^+$Foxp3$^+$) in the colon of mice maintained on a HFD supplemented with or without 5% SA for 4 weeks (n = 10 mice per group). Each dot represents an individual mouse, and the horizontal bars indicate mean values. Statistical significance was assessed by the Welch's *t*-test in (**A**,**C**,**D**), and unpaired Student's *t*-test in (**B**,**E**). n.s., not significant. Cont: HFD diet, SA: HFD diet with 5% sodium alginate.
